# Development of quality indicators for antimicrobial treatment in adults with sepsis

**DOI:** 10.1186/1471-2334-14-345

**Published:** 2014-06-20

**Authors:** Caroline MA van den Bosch, Marlies EJL Hulscher, Stephanie Natsch, Inge C Gyssens, Jan M Prins, Suzanne E Geerlings

**Affiliations:** 1 Department of Internal Medicine, division of Infectious Diseases, Center for Infection and Immunity Amsterdam (CINIMA) Academic Medical Center, Meibergdreef 9, 1105, AZ Amsterdam Zuidoost, The Netherlands; 2 Scientific Institute for Quality of Healthcare (IQ healthcare), Radboud University Medical Center, Nijmegen, The Netherlands; 3 Department of Clinical Pharmacy, Radboud University Medical Center, Nijmegen, The Netherlands; 4 Department of Internal Medicine, Radboud University Medical Center, Nijmegen, The Netherlands; 5 Department of Medical Microbiology and Infectious Diseases, Canisius Wilhelmina Ziekenhuis, Nijmegen, The Netherlands; 6 Hasselt University, Hasselt, Belgium

**Keywords:** Sepsis, Antimicrobial treatment, Quality indicator, Quality improvement, Appropriate antimicrobial use, Appropriate antibiotic use

## Abstract

**Background:**

Outcomes in patients with sepsis are better if initial empirical antimicrobial use is appropriate. Several studies have shown that adherence to guidelines dictating appropriate antimicrobial use positively influences clinical outcome, shortens length of hospital stay and contributes to the containment of antibiotic resistance.

Quality indicators (QIs) can be systematically developed from these guidelines to define and measure appropriate antimicrobial use. We describe the development of a concise set of QIs to assess the appropriateness of antimicrobial use in adult patients with sepsis on a general medical ward or Intensive Care Unit (ICU).

**Methods:**

A RAND-modified, five step Delphi procedure was used. A multidisciplinary panel of 14 experts appraised and prioritized 40 key recommendations from within the Dutch national guideline on antimicrobial use for adult hospitalized patients with sepsis (http://www.swab.nl/guidelines). A procedure to select QIs relevant to clinical outcome, antimicrobial resistance and costs was performed using two rounds of questionnaires with a face-to-face consensus meeting between the rounds over a period of three months.

**Results:**

The procedure resulted in the selection of a final set of five QIs, namely: obtain cultures; prescribe empirical antimicrobial therapy according to the national guideline; start intravenous drug therapy; start antimicrobial treatment within one hour; and streamline antimicrobial therapy.

**Conclusion:**

This systematic, stepwise method, which combined evidence and expert opinion, led to a concise and therefore feasible set of QIs for optimal antimicrobial use in hospitalized adult patients with sepsis. The next step will entail subjecting these quality indicators to an applicability test for their clinimetric properties and ultimately, using these QIs in quality-improvement projects. This information is crucial for antimicrobial stewardship teams to help set priorities and to focus improvement.

## Background

Severe sepsis and septic shock are a substantial burden to health care, affecting millions of patients around the world each year [1]. The average cost of care for a patient with severe sepsis is about 22.000 USD [2]. It is often thought that severe sepsis is primarily seen on the intensive care unit (ICU). However, studies point out that the majority (50 – 68%) of patients with severe sepsis are admitted to a general medical ward [3], with a mortality rate around 26 – 29.5% [4,5]. Since the diagnosis ‘severe sepsis’ is often poorly documented in the medical records by the treating clinicians, specific sepsis-targeted measures may not have been performed and antimicrobial use may have been inappropriate [3].

As the necessary first step in the improvement of appropriate use in patients with sepsis, guidelines have been developed that describe appropriate antimicrobial use in patients with sepsis admitted to a general medical ward or an Intensive Care Unit (ICU). Despite the availability of these guidelines, antimicrobials are used inappropriately: several studies show that inappropriate initial antimicrobial use in patients with severe sepsis or septic shock is associated with a reduction in survival [6-9]. As a second important step towards change and improvement of daily clinical care, the guideline-based development of quality indicators has been suggested [10,11]. Quality indicators (QIs) are measurable elements that can be used to gain insight into the appropriateness of the given antimicrobial treatment, which is important to set priorities and to focus improvement. The aim of our study was to develop a concise and therefore feasible set of QIs to measure and monitor the appropriateness of antimicrobial use in adults with sepsis admitted to a general medical ward and/or ICU.

## Methods

### Delphi survey

The Dutch Working Party on Antibiotic Policy (SWAB) publishes evidence-based guidelines for antimicrobial use. We used the guideline for antimicrobial use in hospitalized patients with sepsis (published online in 2010) as a starting point for the development of a set of QIs [12]. This guideline covers antimicrobial use in all hospitalized adult patients with sepsis, except antimicrobial use in sepsis associated with indwelling intravascular devices that are not removed (tunnelled catheter or totally implantable vascular access devices) and therefore requires different therapy.

**Figure 1:**
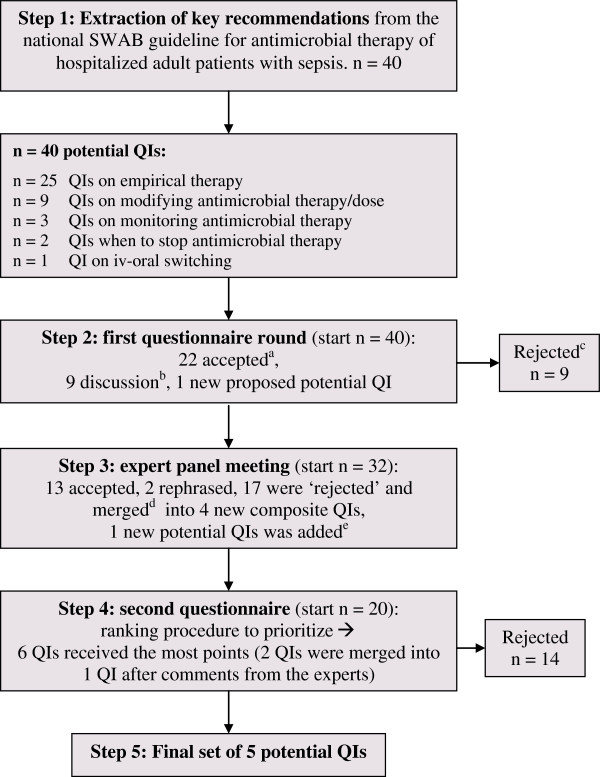
**The step-wise RAND-modified Delphi method. a**. Accepted: the potential QI was selected for the next round because of an overall median score of 8 or 9, without disagreement. Disagreement was defined as the case in which less than 70% of the scores were in the top tertile (scores 7, 8, or 9). **b**. Discussion: the QI had a median score of 7 without disagreement or a median score of 8 or 9 with disagreement, and so it was discussed during the consensus meeting. **c**. Rejected: disagreement between panel members and the median was also lower than 8; the potential indicator was deselected and not discussed during the consensus meeting. **d**. Merged: multiple indicators were ‘rejected’ and merged into a composite, more generic indicator. **e**. Added: the indicator was proposed by one of the experts and was added to the initial set of indicators.

We used a systematic approach -the RAND-modified Delphi method [13,14]- to develop a set of QIs in order to measure the appropriateness of antimicrobial treatment in adult patients with sepsis admitted to a general ward and/or ICU (Figure 1). For developing QIs by means of the Delphi method medical ethical approval was not required.

### Extraction of guideline recommendations

One infectious diseases physician and one quality-of-care specialist independently extracted key recommendations from the national guideline for antimicrobial use in hospitalized patients with sepsis. Disagreements were resolved by consensus. The selected recommendations were translated into potential QIs and each indicator was graded to determine its scientific soundness (level of evidence), using Tables 1 and 2[15].

**Table 1:** **Grading system for methodological quality of individual studies**[15]

	**Intervention**	**Aetiology, prognosis**
**A1**	Systematic review of at least two independent A2-level studies	
**A2**	Randomised Controlled Trial (RCT) of sufficient methodological quality and power	Prospective cohort study with sufficient power and with adequate confounding corrections
**B**	Comparative Study lacking the same quality as mentioned at A2 (including patient-control and cohort studies)	Prospective cohort study lacking the same quality as mentioned at A2, retrospective cohort study or patient-control study
**C**	Non-comparative study	
**D**	Expert opinion	

**Table 2:** Level of evidence of conclusions

	**Conclusions based on**
**1**	Study of level A1 or at least two independent studies of level A2
**2**	One study of level A2 or at least two independent studies of level B
**3**	One study of level B or C
**4**	Expert opinion

### First questionnaire round

The list of the potentially relevant QIs was converted into a written questionnaire and used for the RAND-modified Delphi method to achieve expert consensus on these QIs. The consensus procedure was performed between February and April 2011. All authors of the above mentioned national guideline, and an additional intensive care specialist and a hospital pharmacist were approached, and they all agreed to participate in the multidisciplinary expert panel. Our final expert panel consisted of four infectious diseases physicians (all working primarily outside the ICU), two medical microbiologists, two hospital pharmacists, three intensive care specialists, two haematologists and one general surgeon (14 experts).

The questionnaire was sent by email to the experts, asking them to rate the QIs using the following criteria:

•The potential QI leads to health gain for the patient, less bacterial resistance or promotes efficiency of care;

•The potential QI is generalizable to all adult patients treated for sepsis with antimicrobial use;

•There is enough scientific evidence or expert consensus to justify the recommended care.

The expert panel was asked to rate the potential QIs using a 9-point Likert scale (with 1 denoting “definitely not appropriate care” and 9 denoting “definitely appropriate care”). The answer category ‘cannot assess’ was also available. The panel members (experts) were asked to add suggestions and comments regarding the potential QIs, and also to add additional potential QIs or topics for consideration.

The results from the first questionnaire were analysed using a standardized Microsoft Office Access-based consensus tool. Potential QIs rated with an overall median score of 8 or 9 without disagreement were considered to be face valid and reliable [16], and were accepted as preliminary indicators. Disagreement was defined as the case in which less than 70% of the scores were in the top tertile (scores 7, 8, or 9) [16]. If there was disagreement and the median score was below 8, the potential indicator was rejected and not discussed during the consensus meeting. QIs with a median score of 7 without disagreement or a median score of 8 or 9 with disagreement were discussed during the consensus meeting.

### Expert panel meeting

All panel members were invited for a consensus meeting during which an overview of the first round ratings was provided. Goals of the consensus meeting were to achieve consensus on the QIs with a median score of 7 without disagreement or a median score of 8 or 9 with disagreement, and to rephrase accepted indicators using the comments from the panel in the first round of questionnaires. These QIs and the suggested new QIs by the panel members were discussed and reformulated when necessary.

### Second questionnaire round, ranking procedure

After the consensus meeting, all discussed, reformulated and added potential indicators were included in a second questionnaire. First, the panel was asked whether they agreed (yes or no) with the proposed indicators and their definitions. Redefined indicators were accepted if at least 70% of the experts agreed with the new formulation. Second, the panel was asked to prioritize the potential indicators by selecting the ‘top 5’ of most important indicators. For each number-one ranking by a panel member, we granted a potential QI five points, for each number-two ranking, we granted four points and so on. QIs receiving more than 15% of the maximum possible ranking points were considered to be the most relevant indicators.

## Results

### Extraction of guideline recommendations

Key recommendations were extracted from the national guideline independently by one infectious diseases physician (S.E.G.) and one quality-of-care specialist (M.E.L.J.H.). In consensus, 40 key recommendations were extracted from the national guideline and translated into potential QIs.Figure 1 shows the entire Delphi method as performed in the next steps.

### First questionnaire round

The 40 potential QIs were scored by 12 of the 14 panel members during the first questionnaire round (86% response rate). Twenty-two potential QIs had a high score (8 or 9) without disagreement and were accepted, and nine potential indicators were rejected, see Figure 1 and Table 3. The panel did not agree on nine potential QIs, they either had a median score of 7 without disagreement or a median score of 8 or 9 with disagreement. One new potential indicator regarding adapting the antimicrobial dose to renal function was proposed by one panel member. Results are shown in Table 3.

**Table 3:** Results Delphi procedure: first questionnaire, consensus meeting and second questionnaire

**Quality indicators**	**Level of supporting evidence (see Table**2**)**	**First questionnaire**			**Consensus meeting**	**Second questionnaire**		
		**Median score**	**% in highest tertile**	**Conclusion**		**Nr of experts prioritizing the QI**	**Total score**	**Conclusion**
**1.** Start antimicrobial therapy intravenously in adult patients with sepsis	4	9	92	Accepted^**a**^	Accepted	7	26	Accepted
**2.** Start antimicrobial therapy as soon as possible, preferably within the first hour in adult patients with severe sepsis and septic shock	2	9	100	Accepted	Accepted	12	50	Accepted
**3.** Before starting antimicrobial therapy, at least two sets of blood cultures, and specimens for culture from suspected sites of infection should be taken.	4	9	100	Accepted	Accepted	9	36	Accepted
**4.** For community-acquired sepsis without neutropenia and without an obvious site of infection, start a second or third generation cephalosporin, or amoxicillin and clavulanic acid + an aminoglycoside. Duration of therapy: 7–10 days.	*	8	92	Accepted	Merged^**d**^ into number 43/44			
**5.** For nosocomial sepsis without neutropenia and with no obvious site of infection, start piperacillin with tazobactam, or a second or third generation cephalosporin (except ceftazidime) in combination with either an aminoglycoside or an anti-pseudomonal fluoroquinolone. Duration of therapy: 7–10 days.	*	7	75	Discussion^**b**^	Merged into number 43/44			
**6.** For community-acquired or nosocomial sepsis with neutropenia and without an obvious site of infection, start piperacillin and tazobactam +/− an aminoglycoside or a carbapenem with anti-pseudomonal activity (imipenem/meropenem) as empirical antibacterial regimen. Duration of therapy: 7–10 days.	*	7	75	Discussion	Merged into number 43/44			
**7.** The addition of an aminoglycoside to a beta-lactam agent in adult patients with sepsis is not recommended, unless based on local resistance data and epidemiology (e.g. risk factors for ESBL) a broad spectrum of empirical therapy against Gram-negative pathogens is needed.	*	8	75	Accepted	Accepted	3	6	Rejected
**8.** Glycopeptides should generally not be part of the empirical antibacterial regimen in adults with sepsis (with or without neutropenia), unless patients are known to be colonised with MRSA, or in patients with severe sepsis and neutropenia who received penicillin or cephalosporin prophylaxis.	*	8	83	Accepted	Accepted	0	0	Rejected
**9.** For community-acquired and nosocomial sepsis and prior use of cephalosporins or quinolones within 30 days before presentation, an aminoglycoside should be added or a carbapenem with antipseudomonal activity should be started. This also accounts for adults colonised with ESBL-producing micro-organisms and for those admitted to a hospital with high prevalence of ESBL-producing Enterobacteriaceae. If prevalence is unknown, risk factors for ESBL should be used. Risk factors are: a nosocomial infection, prior use of antibiotics and presence of an indwelling urinary catheter.	2	8	92	Accepted	Accepted	2	4	Rejected
**10.** Empirical antifungal therapy may be considered in selected cases: unexplained sepsis with long-term ICU stay, significant Candida colonisation, and clinical risk factors such as abdominal surgery, anastomosis leakage, the presence of a central venous catheter and the use of broad spectrum antibiotics.	*	8	73	Accepted	Merged into number 43/44			
**11.** For sepsis with a hospital-acquired pneumonia or a ventilated-acquired pneumonia, start amoxicillin and clavulanic acid + an aminoglycoside or ciprofloxacin, or the combination of a second/third generation cephalosporin (excluding ceftazidime) with an aminoglycoside or ciprofloxacin or start piperacillin with tazobactam. Duration of therapy: maximum of 8 days.	1	7	67	Rejected^**c**^				
**12.** For urosepsis, start a second/third generation cephalosporin or the combination of amoxicillin and gentamicin as empirical antibacterial regimen. Duration of therapy: 10 days.	*	7	92	Discussion	Merged into number 43/44			
**13.** For urosepsis and an indwelling urinary catheter, start a second/third generation cephalosporin + an aminoglycoside or quinolone as empirical antibacterial regimen.	*	7	67	Rejected				
**14.** In adults with urosepsis, glycopeptides should be restricted to those septic patients with previously bacteriologically proven *Enterococcus faecium* urinary tract infections in which enterococci are suspected to be the causative pathogens.	*	8	83	Accepted	Merged into number 43^d^			
**15.** For community-acquired intra-abdominal sepsis, start a second/third generation cephalosporin + metronidazole +/− an aminoglycoside or amoxicillin and clavulanic acid +/− an aminoglycoside. Duration of therapy: 5–7 days.	2	8	92	Accepted	Merged into number 43/44			
**16.** For nosocomial intra-abdominal sepsis, start a second/third generation cephalosporin + metronidazole + an aminoglycoside or amoxicillin and clavulanic acid + an aminoglycoside or piperacillin with tazobactam +/− an aminoglycoside. Duration of therapy: 5–7 days.	2	7	75	Discussion	Merged into number 43/44			
**17.** For community-acquired sepsis with cholangitis, start amoxicillin + an aminoglycoside or amoxicillin and clavulanic acid +/− an aminoglycoside. Duration of therapy: up to 3 days following adequate drainage.	*	7	83	Discussion	Merged into number 43/44			
**18.** For nosocomial sepsis with cholangitis, start amoxicillin (with or without clavulanic acid) + an aminoglycoside. Duration of therapy: up to 3 days following adequate drainage.	*	7	75	Discussion	Merged into number 43/44			
**19.** For uncomplicated skin and skin structure infections (SSSI) with sepsis, start flucloxacillin.	2	8	82	Accepted	Merged into number 43			
**20.** For community acquired complicated SSSI with sepsis, start amoxicillin and clavulanic acid. Duration of therapy: 7–10 days.	*	8	67	Discussion	Merged into number 43/44			
**21.** For nosocomial complicated SSSI with sepsis, start amoxicillin and clavulanic acid + an aminoglycoside or piperacillin with tazobactam. Duration of therapy: 7–10 days.	*	8	75	Accepted	Merged into number 43/44			
**22.** For community-acquired sepsis and necrotising fasciitis, start amoxicillin and clavulanic acid + clindamycin.	*	6	50	Rejected				
**23.** For nosocomial sepsis and necrotising fasciitis, start amoxicillin and clavulanic acid + an aminoglycoside + clindamycin or piperacilllin with tazobactam +/− an aminoglycoside + clindamycin.	*	7	67	Rejected				
**24.** Cephalosporins (+/−metronidazole) are suitable alternatives in patients with non-IgE mediated penicillin rash.	*	7	67	Rejected				
**25.** In type I IgE allergic reactions to penicillins, aztreonam or ciprofloxacin +/− an aminoglycoside in combination with vancomycin should be chosen.	*	6	45	Rejected				
**26.** Individualization of dosing using therapeutic drug monitoring should be used whenever possible in adults with sepsis. For aminoglycosides after 3 days and for vancomycin after 5 days.	3	6	50	Rejected				
**27.** When starting vancomycin therapy, at least one trough concentration (just before the fourth dose) should be determined and the concentration should be 15-20 mg/l.	3	7	50	Rejected				
**28.** Frequent measuring of vancomycin trough concentrations is recommended in patients with an increased risk of toxicity or unstable kidney function and > 5 days of treatment.	3	8	75	Accepted	Rephrased to number 47			
**29.** With proven *Pseudomonas* bacteraemia, combination therapy should not be prescribed. Duration of therapy is 7 – 10 days.	2	7	70	Discussion	Merged into number 44/45			
**30.** For sepsis due to methicillin susceptible *Staphylococcus aureus*, start flucloxacillin.	2	8	100	Accepted	Accepted	1	3	Rejected
**31.** Micro-organisms with MICs > 1 mg/l such as *Pseudomonas aeruginosa* or patients with neutropenia should have an intravenous ciprofloxacin dosage of 400 mg tid.	4	8	100	Accepted	Accepted	0	0	Rejected
**32.** Treatment duration should be 14 days for sepsis and pneumonia due to S. *aureus*.	4	9	89	Accepted	Merged into number 44			
**33.** Treatment duration should be 14–21 days for sepsis and pneumonia due to *Legionella pneumophila, Mycoplasma pneumoniae* or *Chlamydia spp*.	4	8	90	Accepted	Merged into number 44			
**34.** Treatment duration should be 14 days in an uncomplicated *Staphylococcus aureus* bacteraemia.	4	9	91	Accepted	Merged into number 44			
**35**. With *S. aureus* bacteraemia it is important to search for complications, this will determine the duration of therapy. Complications are: a secondary infection together with the *S. aureus* bacteraemia (like an endocarditis, infected prosthesis, arthritis, osteomyelitis, meningitis, fasciitis, spleen abscess)	4	8	67	Discussion	Accepted	1	2	Rejected
**36.** Persistence of positive blood cultures for more than 72 hours after starting antibiotics should be considered as complicated *S. aureus* bacteraemia.	4	8	75	Accepted	Accepted	1	1	Rejected
**37.** With sepsis and Listeriosis, the duration of therapy should be 21 days.	4	7	67	Rejected				
**38.** After clinical recovery and when the identity and susceptibility of the causative micro-organism has been determined, a switch to oral agents with high bioavailability should be made.	2	8	91	Accepted	Rephrased to number 48			
**39.** Empirical antimicrobial therapy for presumed sepsis should be discontinued in case of clinical improvement and a lack of clinical and microbiological evidence of infection. Maximum duration of therapy is 7 days.	4	8	83	Accepted	Accepted	2	5	Rejected
**40.** Discontinue broad spectrum antimicrobial therapy after 72 hours of clinical stability in patients with persisting febrile neutropenia that show no clinical or microbiological evidence of infection. Oral antimicrobial prophylaxis against Gram-negative micro-organisms should be continued until resolution of neutropenia.	2	9	91	Accepted	Accepted	2	2	Rejected
**QIs added after first questionnaire:**								
**41.** When starting treatment in adults with sepsis, dose and dosing interval of systemic antimicrobial therapy should be adapted to renal function.	4			Added^**e**^	Accepted	3	4	Rejected
**42.** Concerning empirical therapy for adult patients with sepsis, local guidelines should correspond to the national guideline, but should deviate based on local resistance patterns.	4				Added	9	27	Accepted and merged with number 43
**43.** Empirical antimicrobial therapy (only choice of antimicrobial agent) in all adult patients with sepsis should be prescribed according to the national guideline.	*				Added	2	7	Accepted and merged with number 42
**44.** In all adult patients with sepsis starting antimicrobial therapy the duration of therapy should be prescribed according to the national guideline.	4				Added	3	4	Rejected
**45.** Change empirical antimicrobial therapy to pathogen-directed therapy if culture results become available.	3				Added	7	15	Accepted
**46.** Patients with a *S. aureus* bacteraemia should have a blood culture taken 48 – 72 hours after starting empirical antibiotic therapy.	4				Added	0	0	Rejected
**47.** Therapeutic drug monitoring should be done if vancomycin or aminoglycosides are given > 48 hours, according to the local guideline. The vancomycin trough concentration should be 15-20 mg/l.					Result from rephrasing number 28	1	1	Rejected
**48.** After clinical recovery and when the identity and susceptibility of the causative micro-organism has been determined, a switch to oral agents with high bioavailability should be made. Exceptions are: *S. aureus* bacteraemia, liver abscess, empyema, endocarditis, meningitis and infected prosthetic material.					Result from rephrasing number 38	1	1	Rejected

*Based on available Dutch epidemiology and resistance data. ^**a**^Accepted: the potential QI was selected for the next round because of an overall median score of 8 or 9, without disagreement. Disagreement was defined as the case in which less than 70% of the scores were in the top tertile (scores 7, 8, or 9). ^**b**^Discussion: the QI had a median score of 7 without disagreement or a median score of 8 or 9 with disagreement, and so it was discussed during the consensus meeting. ^**c**^Rejected: disagreement between panel members and the median was also lower than 8; the potential indicator was deselected and not discussed during the consensus meeting. ^**d**^Merged: multiple indicators were ‘rejected’ and merged into a composite, more generic indicator. ^**e**^Added: the indicator was proposed by one of the experts and was added to the initial set of indicators.

### Expert panel meeting

Six panel members (four infectious diseases specialists, one medical microbiologist and one intensive care specialist) were present during the consensus meeting (43%). The 22 accepted QIs with comments from the panel, the nine potential QIs with disagreement and the new indicator were discussed during the meeting.

From these 32 QIs, this smaller panel accepted 13 potential QIs; two QIs were rephrased and one new potential QI was added. The other 17 QIs were rejected and merged into four new composite QIs. These potential QIs had a more generic formulation and contained: starting empirical antimicrobial therapy; duration of therapy; changing empirical therapy to pathogen-directed therapy; and harmonizing local guidelines with the national guideline (indicator 42 – 45, Table 3). This resulted in a total set of 20 potential QIs.

### Second questionnaire round, ranking procedure

After the consensus meeting, the resulting 20 potential indicators were sent to the entire panel for comments and approval. This questionnaire was scored by 13 of the 14 panel members (93% response rate). All 20 indicators were accepted, because in all cases 70% or more of the panel members agreed with the new content and rephrasing. Indicator 42 and 43 (local guidelines should correspond to the national guideline and prescribe according to the national guideline) were merged into one indicator because four out of the 13 panel members (31%) found them to be overlapping indicators. In the same questionnaire the panel members were also asked to rank the QIs. Out of this set of 19 indicators, five indicators received more than 15% of the maximum possible ranking points and were prioritized. They were found to be the most important QIs for antimicrobial care in adult patients with sepsis. The results of the second questionnaire are shown in Table 3 and Table 4 shows the final set of QIs. For the attendance list of the participation of the Delphi procedure and the development of the sepsis guideline, see Additional file 1: Table S1.

**Table 4:** Final set of quality indicators to monitor antimicrobial use in hospitalized adult patients with sepsis

**Indicator number from Table**3	**Quality indicator**	**Numerator description**	**Denominator description**
	**All patients are: hospitalized adult patients with sepsis, severe sepsis or septic shock, where systemic antimicrobial therapy must be started**	**All patients are: hospitalized adult patients with sepsis, severe sepsis or septic shock, where systemic antimicrobial therapy must be started**	**All patients are: hospitalized adult patients with sepsis, severe sepsis or septic shock, where systemic antimicrobial therapy must be started**
Number 1.	Antimicrobial therapy in adult patients with sepsis should be started intravenously.	Number of patients who started with empirical systemic antimicrobial therapy intravenously.	Total number of patients who started with empirical systemic antimicrobial therapy.
Number 2.	Antimicrobial therapy should be started as soon as possible, preferably within the first hour in adult patients with severe sepsis and septic shock.	Number of patients with severe sepsis or septic shock who started with empirical systemic antimicrobial therapy within the first hour after the clinical diagnosis.	Total number of patients with severe sepsis or septic shock, who started with empirical systemic antimicrobial therapy.
Number 3.	Before starting antimicrobial therapy, at least two sets of blood cultures and specimens for culture from suspected sites of infection should be taken.	Number of patients from whom at least 2 blood cultures and specimens for culture from suspected sites of infection were taken before empirical systemic antimicrobial therapy was started.	Total number of patients who started with empirical systemic antimicrobial therapy.
Number 45.	Empiric systemic antimicrobial therapy should be changed to pathogen-directed therapy if culture results become available.	Number of patients with a positive culture and empirical systemic antimicrobial therapy, which was changed to pathogen-directed therapy after the results became available.	Total number of patients with empirical systemic antimicrobial therapy whose culture became positive.
Number 43 and number 42.	Empiric systemic antimicrobial therapy (only choice of antimicrobial agent) should be prescribed according to the national guideline. The local guidelines should correspond to the national guideline, but should deviate based on local resistance patterns.	Number of patients who started with empirical systemic antimicrobial therapy according to the national guideline.	Total number of patients who started with empirical systemic antimicrobial therapy (only choice of antimicrobial agent).
		Number of hospitals with a local guideline that corresponds with the national guideline or only deviates based on local resistance patterns.	Total number of hospitals with a local guideline.

## Discussion

This systematic, stepwise method combining evidence and expert opinion, generated a valid, concise and therefore feasible set of five QIs to measure appropriate antimicrobial use in adult patients with sepsis admitted to general medical wards and/or ICUs. One QI specifically applies to patients with severe sepsis or septic shock (Table 4, indicator 2).

The issue of local resistance patterns and national versus local guideline recommendations for empirical treatment choices was extensively discussed during the Delphi consensus meeting. The national sepsis guideline underlines that hospitals can and should deviate from the recommendations based on local resistance patterns. We therefore favored to follow the national guidelines, but to guarantee the generalizability of the QIs another QI was added during the consensus meeting; local guidelines should correspond to the national guideline, but should deviate based on local resistance patterns (QI number 42).

In a systematic review performed by McGregor et al. empiric or definitive antibiotic therapy was considered to be appropriate if the regimen exhibited in vitro activity against the isolated pathogen(s) [17]. We derived our key recommendations for appropriate antibiotic therapy from the national, evidence-based guideline for antimicrobial use in hospitalized patients with sepsis. This, according to the national and international experts, implies more than correct (empirical or definitive) antibiotic therapy alone: it defines correct antimicrobial use at patient level along the entire antibiotic pathway, from start (including appropriate diagnostics) to streamlining and discontinuing of antimicrobial therapy.

The final set consists of independent QIs, that can be used to provide insight into the appropriateness of current antimicrobial use, to identify where there is room for improvement [18]. In a recent paper by our group [19] we found that patients with urinary tract infections who in particular adherence to the total set of QIs, showed a significant dose–response relationship with a shorter length of hospital stay. This argues for application of the QIs in a bundle approach.

This is the first study that specifically describes the development of QIs for the entire antimicrobial treatment of sepsis patients, also outside the ICU, via the modified Delphi technique. Several studies have described the use of quality measures for antimicrobial sepsis treatment [20-25]. However, some indicators were not systematically developed using a Delphi method [21], and some only focused on optimal sepsis care on the ICU [20,24] or focused on the start of treatment (first 24 hours) and not on the entire clinical course [22,23,25].

The results of our study show resemblance with the concise Surviving Sepsis Campaign Care Bundle originated from the guideline [1,26]. They also defined the optimal start of antimicrobial treatment and taking cultures as important parameters, only our panel members also defined streamlining as an important QI.

Our study has several strengths. We used the systematic modified Delphi method, a common and validated technique in which scientific evidence is combined with expert opinion [13,14,27,28]. Boulkedid and colleagues recently reviewed its use and reporting, and formulated a practical guideline for using this RAND modified Delphi technique. Our procedure is consistent with their guideline [29]. Our panel was multidisciplinary, with 14 experts from 6 different specialties. Furthermore, the response rate of the first and second questionnaire (86% and 93%) was high, which increases the validity of the results.

A limitation of this study is the national setting in which the QIs were developed, with a Dutch national expert panel and a Dutch guideline. This leads to the question of whether the results can be generalized to a wider international population. However, the guideline reviewed and graded the recent international literature and the QI development was performed by a multidisciplinary panel, in which several members have international experience and expertise on the topic.

Another potential limitation was the attendance at the expert panel meeting, which was 43%. However an extensive summary concerning the results from the consensus meeting was sent to all panel members, as they were asked to give their final remarks and approval for the added and rephrased potential QIs. Since 93% returned the second questionnaire, we believe that an incomplete attendance did not undermine the validity of the results.

## Conclusion

We describe the complete and precise development of a concise set of quality indicators for optimal antimicrobial use in hospitalized adult sepsis patients, by means of the Delphi method. This paper can be used as manual for others, since transparency of healthcare becomes more important worldwide, and QIs give insight into the appropriateness of daily clinical care. At this moment, we are testing the applicability of this set of QIs in practice in 22 hospitals. After establishing their clinimetric properties we will analyze the association between adherence to the QIs and outcomes like duration of hospital stay. In the future, our guideline-based indicators can be used for national monitoring of antimicrobial use in hospitalized adults with sepsis or for quality improvement projects. This information is crucial for antimicrobial stewardship teams to help set priorities and to focus improvement.

## Abbreviations

QIs: Quality indicators; SWAB: Dutch working party on antibiotic policy; ICU: Intensive care unit; USD: United States dollar.

## Competing interests

The authors declare that they have no competing interests.

## Authors’ contributions

CB conducted and organized the entire Delphi procedure, analyzed the results, prepared the consensus meeting and drafted the manuscript. MH carried out the extraction of the guideline recommendations, participated in organizing the Delphi procedure and analyzing the results, and rewrote/revised the manuscript. SN participated in the Delphi procedure and rewrote/revised the manuscript. IG participated in the Delphi procedure and rewrote/revised the manuscript. JP participated in the consensus meeting, helped organizing the Delphi procedure and rewrote/revised the manuscript. SE carried out the extraction of the guideline recommendations, participated in organizing the Delphi procedure and analyzing the results, and rewrote/revised the manuscript. All authors read and approved the final manuscript.

## Pre-publication history

The pre-publication history for this paper can be accessed here:

http://www.biomedcentral.com/1471-2334/14/345/prepub

## Supplementary Material

Additional file 1: Table S1 Participation Delphi procedure and development Sepsis guideline. Click here for file
