# Supplementary material for: Development of quality indicators for antimicrobial treatment in adults with sepsis
Source: BMC Infect Dis. 2014 Jun 20;14:345. doi: 10.1186/1471-2334-14-345 (PMC4078010; doi:10.1186/1471-2334-14-345)
Supplement: Additional file 1: Table S1 — Participation Delphi procedure and development Sepsis guideline. [file 1471-2334-14-345-S1.doc]

**Additional file: Table S1.** Participation Delphi procedure and development Sepsis guideline

| First questionnaire round | Consensus meeting | Second questionnaire round | Preparatory committee sepsis guideline |
| --- | --- | --- | --- |
| Prof. I.C. Gyssens, MD, PhD(infectious diseases specialist) | Prof. I.C. Gyssens, MD, PhD(infectious diseases specialist) | Prof. I.C. Gyssens, MD, PhD (infectious diseases specialist) | Prof. I.C. Gyssens, MD, PhD (infectious diseases specialist) |
| H.I. Bax, MD (infectious diseases specialist) | H.I. Bax, MD (infectious diseases specialist) | H.I. Bax, MD (infectious diseases specialist) | H.I. Bax, MD (infectious diseases specialist) |
| S. van Assen, MD, PhD (infectious diseases specialist) | S. van Assen, MD, PhD (infectious diseases specialist) | S. van Assen, MD, PhD(infectious diseases specialist) | S. van Assen, MD, PhD (infectious diseases specialist) |
| C.W. Ang, MD, PhD (medical microbiologist) | C.W. Ang, MD, PhD (medical microbiologist) | C.W. Ang, MD, PhD (medical microbiologist) | C.W. Ang, MD, PhD (medical microbiologist) |
|  | Prof. J.M. Prins, MD, PhD* (infectious diseases specialist) | E.F. Schippers, MD, PhD (infectious diseases specialist) | E.F. Schippers, MD, PhD (infectious diseases specialist) |
| N.P. Juffermans, MD, PhD (intensive care specialist) | N.P. Juffermans, MD, PhD (intensive care specialist) | N.P. Juffermans, MD, PhD (intensive care specialist) |  |
| Prof. M.A. Boermeester, MD (surgeon) |  | Prof. M.A. Boermeester, MD (surgeon) | Prof. M.A. Boermeester, MD (surgeon) |
| J.A. Schouten, MD, PhD (intensive care specialist) |  | J.A. Schouten, MD, PhD (intensive care specialist) | J.A. Schouten, MD, PhD (intensive care specialist) |
| Prof. P. Pickkers, MD (intensive care specialist) |  | Prof. P. Pickkers, MD (intensive care specialist) | Prof. P. Pickkers, MD (intensive care specialist) |
| J.J.W.M. Janssen, MD, PhD (haematologist) |  | J.J.W.M. Janssen, MD, PhD (haematologist) | J.J.W.M. Janssen, MD, PhD (haematologist) |
| Prof. N.M.A. Blijlevens, MD (haematologist) |  | Prof. N.M.A. Blijlevens, MD (haematologist) | Prof. N.M.A. Blijlevens, MD (haematologist) |
|  |  | Y.G. van der Meer, PhD (hospital pharmacist) | Y.G. van der Meer, PhD (hospital pharmacist) |
| P. Sturm, MD, PhD (medical microbiologist) |  | P. Sturm, MD, PhD (medical microbiologist) | P. Sturm, MD, PhD (medical microbiologist) |
| S. Natch, PhD (hospital pharmacist) |  |  |  |

*prof. Prins was a stand-in for dr. Schippers who was unable to attend the meeting on short notice.
